# Successful treatment of non-occlusive mesenteric ischemia in a patient undergoing combined therapy with peritoneal dialysis and hemodialysis: a case report

**DOI:** 10.1007/s13730-026-01139-w

**Published:** 2026-06-15

**Authors:** Takahiko Hoshino, Takahiro Uchida, Minami Koizumi, Tadasu Kojima, Noriko Yoshikawa, Muneharu Yamada, Hiroyuki Higuchi, Toru Sano, Shigeyuki Kawachi, Takashi Oda

**Affiliations:** 1 Department of Nephrology and Blood Purification, Kidney Disease Center, Tokyo Medical University Hachioji Medical Center, 1163 Tatemachi, Hachioji, Tokyo 193-0998 Japan; 2 Department of Digestive and Transplantation Surgery, Tokyo Medical University Hachioji Medical Center, Hachioji, Tokyo Japan

**Keywords:** Dialysis, Non-occlusive mesenteric ischemia, Peritoneal dialysis, Peritonitis

## Abstract

A Japanese man in his 60 s with end-stage kidney disease due to diabetic nephropathy, who initially underwent peritoneal dialysis (PD) and was transferred to combined PD and hemodialysis (HD) therapy 4 years prior, was admitted to our hospital for surgical treatment of lumbar spinal canal stenosis. His rehabilitation continued well after successful surgery; however, he suddenly presented with abdominal pain and cloudy peritoneal dialysate, along with an increased white blood cell count in the peritoneal dialysate effluent, suggesting PD-associated peritonitis. Intraperitoneal administration of antibiotics was ineffective, and contrast-enhanced computed tomography showed areas of poor contrast enhancement and pneumatosis intestinalis in the colon. Emergency laparotomy revealed non-occlusive mesenteric ischemia (NOMI), leading to intestinal resection with ileostomy creation. He made good progress after the surgery and was eventually discharged while receiving thrice-weekly HD. NOMI is a well-known life-threatening condition, and its outcomes in patients receiving dialysis therapy are reportedly unfavorable. Furthermore, to our knowledge, there have been no reported cases of NOMI in patients receiving combined PD and HD therapy. Further accumulation of similar cases is needed to clarify appropriate approaches for treating NOMI in these patients.

## Introduction

Non-occlusive mesenteric ischemia (NOMI), defined as acute intestinal ischemia without structural vascular occlusion, is a well-known serious condition, and its incidence rate among patients receiving hemodialysis (HD) therapy is reportedly higher than that in the general population [1]. The etiology in these patients is presumably associated with hemodynamic changes during HD [1].

Peritoneal dialysis (PD) is generally considered to affect systemic circulatory dynamics to a lesser extent than HD; however, patients receiving PD may still develop NOMI [2]. Furthermore, although data remain limited, it has been reported that the outcome of NOMI in patients receiving PD is also grave [3]. The clinical presentation of NOMI resembles that of PD-associated peritonitis, a more common complication in patients receiving PD, which might disturb prompt recognition of this lethal complication at an early stage, potentially treatable stage.

Herein, we report a case of NOMI in a patient receiving combined PD and HD therapy, a unique type of renal replacement therapy performed in Japan. In the present case, early diagnosis through contrast-enhanced computed tomography and emergency surgery led to a favorable outcome.

## Case report

A Japanese man in his 60 s with end-stage kidney disease due to diabetic nephropathy was admitted to our hospital for surgical treatment of lumbar spinal canal stenosis. He initiated PD 6 years prior and was transferred to combined PD and HD therapy 4 years later due to a decline in residual kidney function. His medical history included coronary artery bypass graft surgery for angina pectoris, hypertension, and type 2 diabetes mellitus; however, he had never experienced PD-associated peritonitis. After the orthopedic surgery was successfully performed on hospital day 2, his physical rehabilitation was going well, and he continued dialysis therapy as usual. There were no episodes of hypotension, massive bleeding, or hemodynamic instability during the perioperative period. HD was performed on hospital day 21 (dry weight, 69.3 kg). His PD prescription was continuous ambulatory PD using a glucose solution (Reguneal LCa 2.5%; Vantive, Tokyo, Japan) and a 7.5% icodextrin solution (Extraneal; Vantive). An ultraviolet light-based sterilization device (Tsunagu; Vantive) was used for dialysate bag changes.

However, on hospital day 27 (postoperative day 25), he suddenly developed diarrhea, followed by cloudy peritoneal dialysate and abdominal pain on hospital day 29. There were no signs of infection at the exit site or along tunnel of the Tenckhoff catheter. At that point, the vital signs were as follows: body temperature, 37.4 °C; blood pressure, 100/62 mmHg; and pulse, 62 beats per minute. Blood tests showed that the white blood cell count was 3,880/µL (neutrophils, 78%) and the C-reactive protein level was 11.64 mg/dL. The white blood cell count in the PD effluent was 1,507 cells/µL (neutrophils, 90%), and a diagnosis of PD-associated peritonitis was made, and intraperitoneal administration of ceftazidime and vancomycin was initiated. Blood cultures were positive for *Clostridium perfringens*, whereas the PD effluent was culture-negative.

**Fig. 1:**
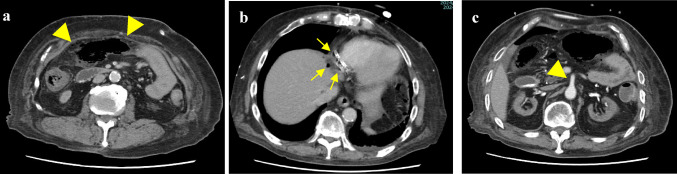
Contrast-enhanced computed tomography image of the patient. Yellow arrowheads in panel (**a**) point to poor contrast enhancement and pneumatosis intestinalis in the ascending and transverse colon. In panel (**b**), the yellow arrows point to portal venous gas. In panel (**c**), the yellow arrowhead points to the superior mesenteric artery, which shows preserved contrast enhancement

**Fig. 2:**
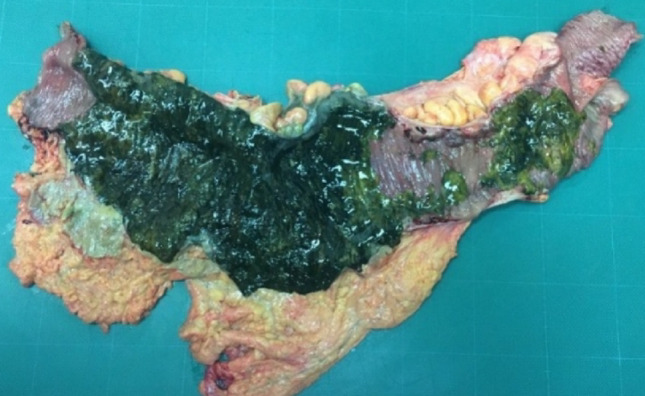
Macroscopic findings of the surgical specimen. Widespread necrosis extending from the ascending colon to the transverse colon necessitated surgical resection. No bowel perforation was observed in the patient

Despite antibiotic therapy, his general condition worsened, and his next HD therapy scheduled on hospital day 28 was postponed. Subsequently, on hospital day 30, he developed severe diffuse abdominal pain with rebound tenderness and muscular defense. Blood tests on hospital day 30 demonstrated that inflammatory markers were further increased (white blood cell count, 8,340/µL (neutrophils, 86.1%); C-reactive protein, 27.91 mg/dL; Table 1). His body weight was 68.1 kg, suggesting dehydration. Contrast-enhanced computed tomography (CT) revealed areas of poor contrast enhancement and pneumatosis intestinalis in the ascending and transverse colon (Fig. 1a) and hepatic portal venous gas (Fig. 1b), whereas contrast enhancement of the mesenteric arteries was maintained (Fig. 1c), with mild calcification only in the periphery of the superior mesenteric artery. Emergency laparotomy was performed, and bowel necrosis extending from the ascending colon to the transverse colon was observed, necessitating surgical resection of the ileocecal region and the ascending and transverse colon (Fig. 2). Histopathological examination of the resected bowel revealed extensive mucosal erosion and ulceration with acute inflammatory cell infiltration, consistent with ischemic bowel injury. There was no bowel perforation, and a final diagnosis of NOMI was made. An ileostomy was created, and the Tenckhoff catheter was removed during the operation. There were no signs of encapsulating peritoneal sclerosis, and a peritoneal biopsy was not performed intraoperatively. Antibiotic therapy was switched to intravenous tazobactam/piperacillin and subsequently to ampicillin/sulbactam after the abdominal surgery. The patient made good progress while transitioning to thrice-weekly HD, because there was a high risk of intra-abdominal complications, including infection and intestinal adhesions. The patient was finally discharged on hospital day 68.

**Table 1:** Laboratory data of the patient on hospital day 30

Hemoglobin	11.6 g/dL	Uric acid	5.0 mg/dL
Red blood cell	3.78 × 10^6^/μL	Blood urea nitrogen	53.0 mg/dL
Hematocrit	36%	Creatinine	8.99 mg/dL
White blood cell count	8340/μL	Sodium	130 mEq/L
Neutrophils	86.1%	Chloride	96 mEq/L
Eosinophils	0.3%	Potassium	3.4 mEq/L
Basophils	0.1%	Calcium	7.3 mg/dL
Lymphocytes	6.8%	Phosphate	3.2 mg/dL
Monocytes	5.9%	Glucose	80 mg/dL
Platelet count	16.5 × 10^4^/μL	C-reactive protein	27.91 mg/dL
Total protein	5.4 g/dL	Procalcitonin	70.3 ng/mL
Albumin	2.0 g/dL	Venous gas pH	7.373
Total bilirubin	0.2 mg/dL	pCO_2_	34.9 mmHg
AST/ALT	11/7 U/L	Bicarbonate	20.8 mmol/L
LDH	178 U/L	Base excess	-3.9 mmol/L
ALP	92 U/L	Lactate	23 mg/dL

pCO_2_, partial pressure of carbon dioxide; AST, aspartate aminotransferase; LDH, lactate dehydrogenase; ALP, alkaline phosphatase

## Discussion

Since it was first described by Ende in 1958 [4], the comprehensive pathophysiology of NOMI has yet to be elucidated; however, mesenteric vasoconstriction, which is triggered by a variety of factors, including intestinal hypoxia or ischemia–reperfusion injury, cardiovascular stress, or infection, has been suggested to play a crucial role [5]. Although rare in the general population, with an incidence of 0.006–0.047 cases per 100 persons/year, its incidence is much higher in patients receiving HD; the reported incidence is 2.29 cases per 100 patients receiving HD per year [1]. Several risk factors for NOMI have been identified in patients receiving HD, such as older age, diabetes mellitus, atherosclerosis, and HD vintage [6], of which intradialytic hypotension is considered the greatest contributing factor [7]. Similar to the present case, the cecum was the most commonly affected segment of the bowel in NOMI. This is because during hypoperfusion, the steal phenomenon affects the superior mesenteric artery, the source of the ileocolic artery, thereby leading to ischemia in the cecum and ascending colon [7, 8].

Contrary to its high incidence rate among patients receiving HD, NOMI has rarely been reported in patients receiving PD, presumably reflecting the lower incidence of hypotensive episodes in these patients [2]. However, many patients receiving PD have risk factors for NOMI [9], and it has also been reported that PD dialysate could remove endogenous aldosterone, which is necessary for proper sodium absorption in the gastrointestinal tract [2]. A literature search using PubMed found 12 cases of NOMI in patients receiving PD [2, 3, 8, 10–14] (Table 2); however, to our knowledge, the present case is the first reported case of NOMI in a patient receiving combined PD and HD therapy. Combined PD and HD therapy, a unique type of renal replacement therapy performed in Japan, reportedly brings favorable clinical outcomes, presumably because effective fluid management can be achieved by once-weekly HD [15, 16]. On the other hand, daily PD could aggravate a dehydrated state if precipitating factors such as diarrhea exist. Furthermore, although NOMI is a relatively well-known complication in patients receiving HD alone, it has rarely been reported in patients receiving PD. Therefore, while it is expected that the incidence in patients receiving combined HD and PD therapy would fall somewhere between the incidences of patients receiving either HD or PD, the lack of reports to date makes it unclear whether the condition is truly extremely rare or whether it occurs but has simply not been reported. There is another important pitfall; the clinical presentation of NOMI resembles that of PD-associated peritonitis, a more common complication in patients receiving PD. We considered that these several factors could contribute to the onset of NOMI and its delayed diagnosis.

**Table 2:** Summary of the reported cases of non-occlusive mesenteric ischemia in patients receiving peritoneal dialysis therapy

Author (Year)	Age	Sex	Primary disease	PD vintage	Necrotic area	Outcome
Oda Y et al. [8]	70	F	Diabetic Kidney Disease	10 Months	Terminal ileum and right colon	Survival
Vishwakarma K et al. [10]	62	F	Diabetic Kidney Disease	6 Years	Jejunum to transverse colon	Deceased
Vishwakarma K et al. [10]	70	M	Diabetic Kidney Disease	5 Years	Small intestine	Deceased
Yu CC et al. [11]	N.D	N.D	N.D	N.D	N.D	Survival
Yu CC et al. [11]	N.D	N.D	N.D	N.D	N.D	Deceased
Yu CC et al. [11]	N.D	N.D	N.D	N.D	N.D	Deceased
Archodovassilis F et al. [3]	76	F	Nephrosclerosis	5 Years	Ascending to sigmoid colon	Deceased
Liu HL et al. [2]	71	F	Diabetes	14 Months	Jejunum to transverse colon	Deceased
Liu HL et al. [2]	40	M	Unknown	7 Years	Small intestine	Deceased
Korzets Z et al. [12]	72	F	Polycystic kidney disease	12 Months	Ascending and transverse colon	Deceased
Appel RG [13]	48	F	Membranoproliferative glomerulonephritis	2 Years	Ascending and transverse colon	Deceased
Schulak JA et al. [14]	67	F	Diabetic Kidney Disease	9 Months	Small intestine	Deceased
Our case	60	M	Diabetic Kidney Disease	6 Years	Ascending and transverse colon	Survival

N.D. Not described

In the present case, which had multiple risk factors such as diabetes mellitus and atherosclerosis, it is plausible that NOMI was the primary event, leading to a disruption in the mucosal barrier and a resultant bacterial translocation (*Clostridium perfringens* bacteremia). Based on this interpretation, and given that no bacteria were detected in the PD effluent, it is possible that the peritonitis-like findings initially observed in this case reflected non-infectious inflammation of the peritoneum caused by intestinal ischemia. Although the possibility might be lower, there is another hypothesis that intestinal infection with *Clostridium perfringens* led to endogenous peritonitis, and the resulting dehydrated state caused NOMI; his body weight was far below his dry weight when he developed NOMI. The PD effluent of our patient was culture-negative; however, about one-fifth cases of PD-associated peritonitis are reportedly culture-negative, clearly needing more sensitive and accurate diagnostic methods [17].

As shown in Table 2, the prognosis is generally poor for patients receiving PD who develop NOMI. This is probably due to delayed recognition of this condition at an early treatable stage because the clinical presentation of NOMI resembles that of PD-associated peritonitis, a more common complication in patients receiving PD. Imaging tests using contrast agents tend to be avoided in patients receiving PD because of concerns about further decline in residual kidney function, which could also prevent precise diagnosis at an appropriate stage. In our patient, the initial abdominal symptoms on hospital day 29 were mild; there were only cloudy peritoneal dialysate and abdominal pain without signs of peritoneal irritation. We therefore did not perform contrast-enhanced CT at that point, but the patient developed severe diffuse abdominal pain with rebound tenderness and muscular defense on day 30. Because of these severe manifestations, we considered it urgent to evaluate the necessity of abdominal surgery, and performed a contrast-enhanced CT scan. Our patient had a good outcome owing to early diagnosis through contrast-enhanced computed tomography and emergency surgery, although the timing of NOMI onset (hospital day 30) was far different from that of HD therapy (hospital day 21), making it difficult to promptly differentiate this condition. Further accumulation of similar cases is required to derive appropriate approaches for treating this life-threatening condition.
